# Model and Method for Providing Resilience to Resource-Constrained AI-System

**DOI:** 10.3390/s24185951

**Published:** 2024-09-13

**Authors:** Viacheslav Moskalenko, Vyacheslav Kharchenko, Serhii Semenov

**Affiliations:** 1 Department of Computer Science, Sumy State University, 116, Kharkivska Str., 40007 Sumy, Ukraine; 2 Department of Computer Systems, Networks and Cybersecurity, National Aerospace University “KhAI”, 17, Chkalov Str., 61070 Kharkiv, Ukraine; v.kharchenko@csn.khai.edu; 3 Cyber Security Department, University of the National Education Commission, Ul. Podchorążych 2, 30-084 Kraków, Poland; serhii.semenov@up.krakow.pl

**Keywords:** dynamic deep neural networks, affordable resilience, robustness, adversarial attack, fault injection, concept drift

## Abstract

Artificial intelligence technologies are becoming increasingly prevalent in resource-constrained, safety-critical embedded systems. Numerous methods exist to enhance the resilience of AI systems against disruptive influences. However, when resources are limited, ensuring cost-effective resilience becomes crucial. A promising approach for reducing the resource consumption of AI systems during test-time involves applying the concepts and methods of dynamic neural networks. Nevertheless, the resilience of dynamic neural networks against various disturbances remains underexplored. This paper proposes a model architecture and training method that integrate dynamic neural networks with a focus on resilience. Compared to conventional training methods, the proposed approach yields a 24% increase in the resilience of convolutional networks and a 19.7% increase in the resilience of visual transformers under fault injections. Additionally, it results in a 16.9% increase in the resilience of convolutional network ResNet-110 and a 21.6% increase in the resilience of visual transformer DeiT-S under adversarial attacks, while saving more than 30% of computational resources. Meta-training the neural network model improves resilience to task changes by an average of 22%, while achieving the same level of resource savings.

## 1. Introduction

### 1.1. Motivation

The proliferation of Artificial Intelligence (AI)-enhanced edge computing devices, such as smart sensors, drones, autonomous vehicles, and intelligent industrial controllers, is reshaping technological landscapes. However, for safety-critical applications, ensuring resilience is paramount. Beyond traditional information system security and robustness, specific resilience considerations applicable to AI are necessary. AI systems can be vulnerable to various disruptions including adversarial attacks, faults, concept drifts, and out-of-distribution data.

Resilience to these disturbances is generally achieved through various protective mechanisms that typically require or consume excessive resources. In the context of edge computing, resources are notably limited. Some researchers have been exploring the trade-offs between resilience and resource efficiency in AI systems. Approaches such as adversarial training, fault-aware training, and domain generalization, in conjunction with techniques like compression, pruning, and knowledge distillation, have been employed to provide affordable resilience.

The ongoing challenge is to maintain functional efficiency while enhancing resilience and efficiently managing resources. Potential solutions may lie in striking a balance and implementing adaptive computing strategies to accommodate resource limitations and the need to counter disruptions. While there are studies that address individual aspects of resilience and resource-efficient usage, the issue of ensuring rational resilience in AI systems under resource constraints remains underexplored.

These methodological gaps underscore the urgency for research and development of AI systems that harmonize and implement resilience components in a resource-efficient manner, thereby mitigating the impact of various kinds of perturbation on AI performance. This calls for innovative frameworks that not only address current limitations but also anticipate future challenges in deploying resilient AI systems in resource-constrained environments.

### 1.2. State-of-the-Art

Popular methods and frameworks for deploying AI systems on resource-constrained devices are considered in [1,2]. These methods primarily focus on accelerating inference through weight quantization, weight pruning, and knowledge distillation. However, the resulting models are not optimized for resilience to disturbances.

Sources of destructive impacts on AI systems and protection methods against them are discussed in [3,4]. A widely used approach includes applying denoising autoencoders, generative adversarial networks, and denoising diffusion probabilistic models to mitigate noise in input data caused by adversarial perturbations [5,6,7]. These methods do not provide noise reduction simultaneously in the spatial domain, frequency domain, and latent space. Moreover, the implementation of these methods requires increased resource consumption both during the training phase and at inference time. A common approach to enhancing resilience against network weight corruption is ensembling and incorporating duplicate neurons. This significantly increases resource consumption during both the training and adaptation phases, as well as during inference.

Vulnerability of ReLU, Sigmoid, and Tanh activation functions to adversarial attacks and weight corruption was investigated in [8,9]. In addition, it is widely known that the Sigmoid and Tanh activation functions have a problem with saturation at extreme values, which leads to gradient vanishing and reduces the model’s ability to adapt to attacks during the fine-tuning stage. Enhancing resilience to weight damage and adversarial attacks by improving activation functions is examined in [10,11]. For instance, Relu6 reduces the attack surface by limiting the maximum value of the activation function, while the Leaky ReLU activation function enhances network adaptation efficiency and speed by providing more informative gradients.

Various approaches to adversarial training to increase adversarial robustness are discussed in [12,13]. The regularization effect of fault-aware training to enhance system fault tolerance is investigated in [11,14]. However, the issue of rapid adaptation to disturbances has not been fully explored. Test-time adaptation methods, where each input sample is used to assess marginal uncertainty and fine-tune the network, are examined in [15]. Methods of meta-learning to accelerate adaptation are discussed in [16,17]. Parameter-efficient transfer learning methods to reduce resource costs during model fine-tuning for new tasks or conditions are explored in [18,19].

Ideas and methods of dynamic neural networks as an effective approach to reducing computational complexity through input-dependent computations are considered in [20,21]. Various versions of early exit architectures are explored in [20]. Different versions of neural network block disabling using trainable gate modules are investigated in [21]. Gate units are more versatile as they can activate and deactivate various parts of the network, not just the top layers. Dynamic switching depends on the characteristics of the input data, but the impact of destructive disturbances is not considered, which can impair the resilience of the AI system. Dynamic early exit architectures and their robustness to adversarial attacks are investigated in [22]. The study in [23] proposed a system parameter optimization method which takes into account resource limitations. The method is based on the search for a compromise between the efficiency criterion computed under normal conditions and the integral resilience system indicator calculated under the influence of disturbances. The fault tolerance of dynamic early exit architectures is explored in [24].

However, there is still a lack of studies considering a broader concept of resilience for the more general case of dynamic neural networks based on trainable gate modules.

### 1.3. Objectives and Contributions

The aim of this research is to develop a model and method of ensuring resilience for a resource-constrained AI system, which would function normally, despite destructive disturbances.

An AI system is considered resilient to a certain type and level of disturbances if it demonstrates robustness, rapid recovery, and performance improvement when disturbed. A smaller decline and a faster recovery of performance metrics under the influence of disturbances are attributes of a more resilient system. If the permissible level of performance metrics decline, and the allowable number of iterations for performance recovery are known, the AI system is deemed resilient to a given disturbance if its performance metrics remain within the specified boundaries.

The key objectives are as follows:

- To develop a new resource-efficient model and a training method, which simultaneously implement components of resilience such as robustness, fast recovery, and improvement;
- To test the model’s and training method’s ability to provide robustness, fast recovery, and improvement.

Structurally, the work consists of the following sections. A new architecture of an AI system for ensuring resilience in resource-constrained environments based on dynamic neural networks is presented in Section 2. Section 3 describes a new training method for ensuring resilience in resource-constrained environments based on meta-learning from adaptation to perturbation, with subsequent tuning on newly labeled and unlabeled observations. Section 4 describes the experimental results of testing the proposed model and method for training an AI system on the example of the image classification task using backbones from convolutional networks or visual transformers. The research results are discussed in Section 5. Section 6 concludes the paper and describes the directions of future research.

The main contribution of the research includes a set of proposed principles, a model, and a training method which provides resource-efficient resilience capabilities to fault injections, concept drifts, and adversarial attacks. In addition, we investigate the dependence of the resilience of a dynamic convolutional network and dynamic visual transformer on the dynamic compression rate with different pre-training methods. Pre-training on training data under normal conditions, pre-training under disturbances, and meta-pretraining on the results of adaptation to disturbances are considered.

The novelty of the model lies in the use of the LeakyReLU6 function in a dynamic neural network and the change in the network structure, not only during forward pass but also during backward propagation. The novelty of the training method lies in the use of offline meta-pretraining on the results of adaptation to disturbances of a dynamic neural network and online adaptation on a small number of labeled and unlabeled examples. In this case, online tuning is performed only for those modules that were activated during the forward pass.

## 2. Architecture of Resource-Efficient and Resilient AI-Model

### 2.1. Principles

Proposed neural network architecture which can provide acceptable resilience under resource constraints is designed on the following principles:

- Combining ReLU6 and LeakyReLU activation functions for efficient training and inference under conditions of data and neural network weight perturbations;
- Dividing the model into blocks (e.g., several convolutional layers, multi-head self-attention, feed-forward network), and adding skip-connections that bypass each block and gate modules that can disable blocks depending on the input context;
- Gate modules should be significantly computationally simpler than the model blocks they switch.

The main idea behind the implementation of these principles is to make small changes and architectural add-ons that absorb part of the disturbances and save resources by adaptively activating the relevant blocks of the neural network. In this case, the architectural add-on consists of the implementation of a dynamic neural network. The network’s gates can also be attacked, but since they are trained under disturbances together with the main network, the overall resilience increases.

For the simplicity of the experiments, we consider the case of processing information in the inference mode using a mini-batch size of 1, since the computation graph changes for each sample, which complicates parallel processing with mini-batches. This issue is mitigated in the scenario of edge computing, where the input signal by itself is sequential and the computing hardware is less powerful than high-end platforms.

### 2.2. Selected Architectures

**Figure 1:**
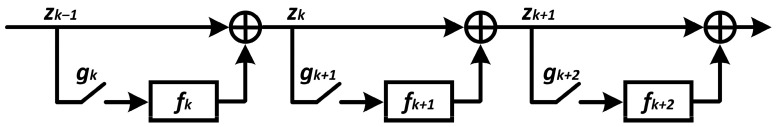
General architecture of dynamic neural network.

The proposed architecture of the dynamic neural network backbone is shown in Figure 1. Depending on the input data, a certain group of blocks is activated to calculate the relevant features [25]. It is assumed that, with a proper training methodology, the subset of layers or blocks that will provide the most reliable forecast under the influence of disturbances will be activated in the inference mode.

The dependence between the input tensor $z_{k-1}\;$ and the output tensor $z_{k}$ of the *k*-th block with the corresponding skip connection and gate at training time can be defined as follows

(1) $$z_{k}=z_{k-1}+g_{k}\left(z_{k-1}\right)f_{k}\left(z_{k-1}\right),$$

where $g_{k}$ is the gate function, $g_{k}\;\epsilon \;\left\{0,\;1\right\}$;

$f_{k}$ is a function of calculating the features of the k-th structural block of a convolutional network (CNN) or visual transformer (ViT), for example, ResBlock without residual connection, multi-head self-attention without residual connection, feed-forward network without residual connection, and other similar ones.

The computational graph for computing $z_{k}$ at inference time can be defined as follows

(2) $$z_{k}=\left\{\begin{array}{c} z_{k-1},\;\;\;\;\;\;\;\;\;\;\;\;\;\;\;\;\mathrm{if}\;g_{k}\left(z_{k-1}\right)=0\\ z_{k-1}+f_{k}\left(z_{k-1}\right)\;\;\mathrm{if}\;g_{k}\left(z_{k-1}\right)=1 \end{array}\right..$$

**Figure 2:**
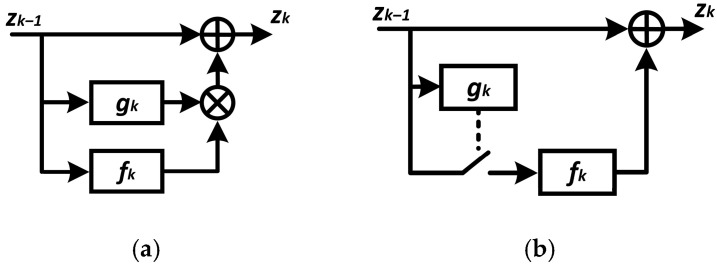
Schematic illustration of the structural unit of a dynamic neural network with one skip-connection: (**a**)—training mode; (**b**)—inference mode.

Figure 2 illustrates expression (1) and expression (2) in schematic form. All feature extraction layers and gate units are computed in the training mode, and all gate units but not all feature extraction layers, are computed in the inference mode.

**Figure 3:**
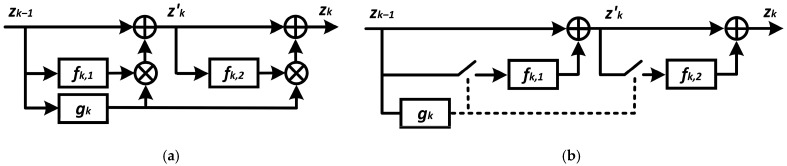
Schematic illustration of the structural unit of a dynamic neural network with two skip-connectors: (**a**)—training mode; (**b**)—inference mode.

Gates can skip several consecutive layers if they form a single structural unit, but each has a separate skip connection (Figure 3) [26]. This is useful for transformers whose encoders contain multi-head self-attention (MSA) layers and a multilayer perceptron (MLP) layer with separate skip connections.

Based on the functional purpose of the gate unit and the specifics of training multilayer neural networks, it should have the following properties [25,26]:

- Low computational complexity compared to the building block that is activated or deactivated;
- Stochasticity to prevent the mode from decaying into trivial decisions, such as always or never executing a block;
- The ability to generate discrete solutions and calculate gradients to optimize the parameters of the gate unit.

**Figure 4:**
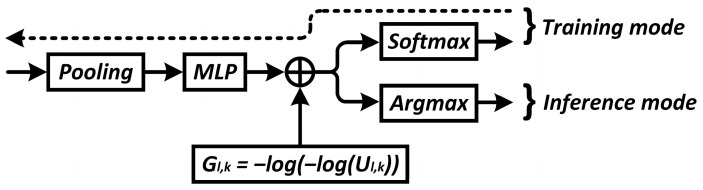
The architecture of the gate unit.

Figure 4 shows the structure of the gate unit in the training and inference modes. In this figure, the operator ⊕ denotes element-wise summation, while ⊗ represents element-wise multiplication. The addition of Gumbel noise G = −log(−log(U)), where U∼Unif [0, 1], to the neural output of the gate unit allows us to add some stochasticity to avoid trivial solutions in the inference mode. The use of the Gumbel-Softmax trick ensures the differentiation of the gate unit and the ability to optimize its parameters.

**Figure 5:**
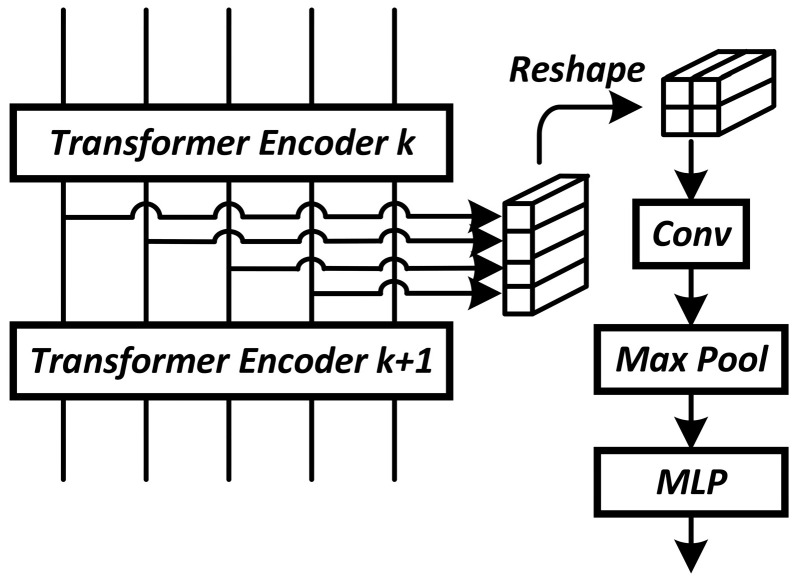
Connecting the gate unit to the input of the transformer encoder.

In the experiments, the gate unit model is the same for all network blocks. MLP with one hidden layer of 24 neurons and an output layer of 2 neurons is used to calculate the relevance features of blocks in the gate unit. The activation function used is the LeakyReLU6 described above. In the case of a convolutional network, the pooling function can be implemented as global average pooling. In the case of the visual transformer, the sequence of token vectors is first reshaped into a 2D grid similar to the intermediate representation of CNN, followed by convolution (16 filters with a 3 × 3 kernel) i Max Pool 2 × 2 (Figure 5) [27].

## 3. Training Method Design

### 3.1. Principles

Resilience-aware AI training with limited resources is proposed to be performed in two stages. The first, preparatory stage should be performed in an environment without significant resource or time constraints. This stage can be repeated periodically if a significant amount of new training data is accumulated. The second, fine-tuning stage is designed to adapt and absorb disturbances of various kinds, and can be performed on data with or without labels (or feedback).

The implementation of the preparatory stage of training a resilient AI system is based on the following principles [28]:

- Simultaneous training of the main network weights and the weights of the gate units;
- Training is performed first on the main training set under normal conditions, and then performed as episodic few-shot learning tasks with adaptation to each type of synthetic perturbation;
- Generation of synthetic perturbations of data or weights according to the white box scenario;
- Implementing generation of perturbations, such as novelty or drift of concepts, by changing tasks;
- Generalization of experience during the preparatory phase should be based on meta-updating using the MAML, REPTIL algorithm or meta-free weight averaging;
- The loss function of the neural network model should contain a component that characterizes the complexity of calculations or the degree of deviation from the desired level of network compression.

The implementation of the fine-tuning stage for a resilient AI system in an environment with constrained resources is based on the following principles:

- Only those blocks that were activated during the forward pass are corrected, so you need to save the output of each gate after forward passing;
- Unlabeled images on which the model has a significant predictive uncertainty can be used for test-time adaptation.

The main idea behind the implementation of these principles is that the preparatory stage of training should prepare the AI system for the impact of disturbances and operating with limited computing resources. The preparatory stage should ensure the system’s ability to absorb disturbances and quickly adapt to unabsorbed disturbances with minimal resource costs.

### 3.2. Methods of Ensuring the Resilience of Resource-Constrained AI-System

**Figure 6:**
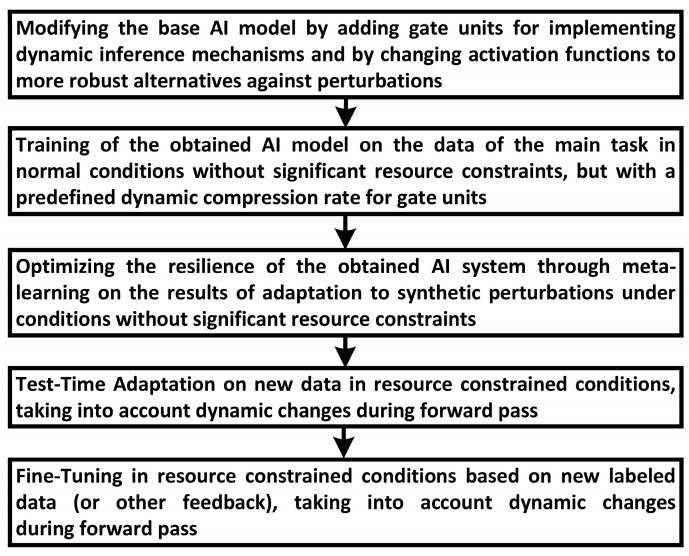
Stages of optimization of resilient AI system parameters in resource-constrained environments.

Ensuring the resilience of the AI in resource-constrained conditions requires the optimization of the main network and gate models so that only those components of the neural network that can provide the most accurate forecast under the influence of disturbances are computed in the inference mode. In addition, the process of training the neural network on a limited number of labeled and unlabeled samples should make it adaptable to changing tasks and conditions. Taking into account the principles described in Section 3.1, the method of ensuring the resilience of the AI system consists of the implementation of four main stages of training (Figure 6).

Let $\left\{\tau_{i}|\;i=\bar{1,N}\right\}$ denote a set of disturbance implementations for AIS. Disturbances $\tau_{i}$ can represent adversarial attacks, fault injections, or switching to a new task. Let $D_{\mathrm{base}}=\left\{D_{\mathrm{base}}^{\mathrm{tr}};D_{\mathrm{base}}^{\mathrm{val}}\right\}$ denote a dataset on which the model was trained to perform the main task under known conditions. The morel is also given a dataset $D=\{D_{k}^{\mathrm{tr}};D_{k}^{\mathrm{val}}|k=\bar{1,K}\}$ for K few-shot learning tasks, where fine-tuning data $D_{k}^{\mathrm{tr}}$ is used in the fine-tuning stage and validation set $D_{k}^{\mathrm{val}}$ is used in the meta-update stage. There is also a given set of parameters $\Psi =\langle \theta ,\phi \rangle$, and W, where θ are parameters of base AI model backbone, ϕ are parameters of gate units, and W are task-specific parameters (model head parameters). Head weights $W_{\mathrm{base}}$ for the main task should be trained on $D_{\mathrm{base}}$.

Gradient-based meta-learning requires finding values of the parameters $\Psi^{*}$ which will ensure the minimum expected loss function L calculated on the set of implementations of different types of disturbance $\tau_{i}$ during its adaptation on $D_{\tau_{i}}$.

(3) $$\Psi^{*}=\arg\underset{\Psi }{\min}\underset{\tau_{i}\sim p\left(\tau \right)}{E}\left[L_{\tau_{i}}\left(U_{\tau_{i}}\left(\Psi ,W_{\tau_{i}},D_{\tau_{i}}\right)\right)\right]$$

where U is an operator which combines a disturbing influence and adaptation in T steps, mapping the current state of Ψ to new state of Ψ.

**Figure 7:**
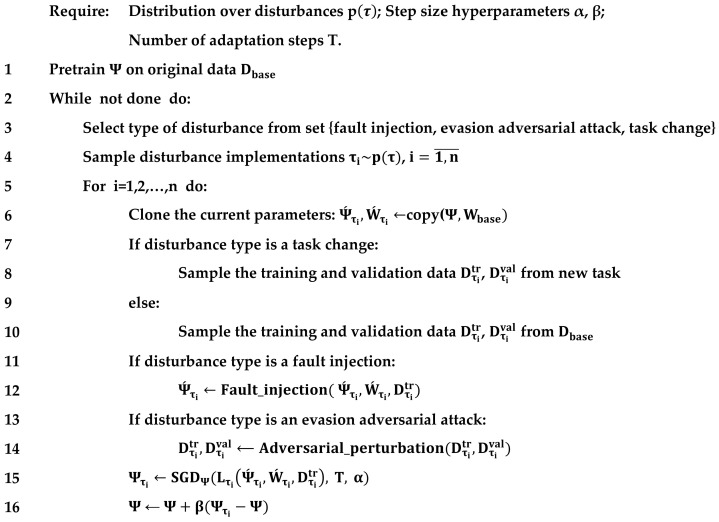
Pseudocode of meta-learning for AIS resilience optimization.

The pseudo-code of the meta-learning algorithm for increasing the resilience of AIS is shown in Figure 7. Stochastic gradient descent (SGD) algorithm with T steps in the U operator performs the gradient meta-update Ψ. To simplify computations and increase stability, meta-tunable parameters can be updated using the REPTIL algorithm [29]. The type of disruptive influence does not change within a single meta-adaptation step. However, each meta-adaptation step begins with the selection of a disruptive influence type, followed by the generation of n implementations of the disruptive influence with a subsequent nested adaptation loop for each of them.

As shown in Figure 7, the formation of adversarial samples is implemented by the Adversarial_perturbation() function. It is proposed to use white box attacks, for example FGSM attacks or PGD attacks, for meta-learning [30]. Black box attacks, such as attacks based on the search algorithm of the covariance matrix adaptation evolution strategy (CMA-ES), are proposed for testing [31]. The level of perturbation is limited by the $L_{\infty }$-norm or $L_{0}$-norm. In this case, if the image is normalized by dividing pixel brightness by 255, the specified disturbance level is subsequently divided by 255 as well.

The Fault_injection()  function generates fault injections to affect the neural network tensors [32]. It is suggested that the most difficult fault type is chosen to absorb, which involves generating an inversion of a randomly selected bit (bit-flip injection) in the weight coefficient of the model. During training, it is suggested to damage the most sensitive weights. To determine such weights, test datasets should be passed through the network with gradients calculated and sorted by their absolute values. In the top-k weights with the highest gradient, one bit can then be inverted randomly. It is proposed that damage to random weights for testing purposes is generated.

Changing tasks during meta-learning increases the speed of adaptation to drift and novelty. Forming a sample of other tasks can be done by randomizing the domain of the same task, or by selecting tasks from relevant domains but sampling truly different tasks. These two approaches can also be combined. In any case, an attempt should be made to sample data from larger and more diverse sets than $D_{\mathrm{base}}$.

To ensure the resource efficiency of a trained AI system in different operating conditions, its learning loss function should include both the component of the main task and the component related to the amount of available computational resources. The simplest AI task, i.e., classification of observations, is considered in experiments below. The main component of the loss function can be a cross-entropy function $L_{\mathrm{CE}}$. The Lusage function, in turn, is used to calculate the deviation of the desired dynamic compression rate of the neural network from the actual rate. This function is affected by the number of activated blocks of the main model based on decisions made by the gate units. In this case, the loss function takes the following form [21,26]:

(4) $$L=L_{\mathrm{CE}}+{\lambda L}_{\mathrm{usage}}$$

where λ balances the two losses (λ=2 in our experiments).

The $L_{\mathrm{usage}}$ function is calculated in a similar way to [20], where the target rate of switching on each neural network unit is given

(5) $$L_{\mathrm{usage}}=\frac{1}{K}\sum_{k=1}^{K}{\left(\frac{1}{n}\sum_{i=1}^{n}g_{k,i}-t_{k}\right)}^{2},$$

where $g_{k,i}$ is the output of the k-th gate for the i-th training data instance;

$t_{k}$ is the approximated target rate of execution of each neural network block on a data mini-batch, $t_{k}\in \left(0,1\right)$ ($t_{k}=0.5$ by default);

K is the number of gate units that control the activation of K neural network blocks;

n is the size of the training mini-batch.

Both fine-tuning on newly labeled samples and test-time adaptation are performed without using $L_{\mathrm{usage}}$. It is assumed that when adapting to new functional conditions, we reuse the dynamic mechanism trained on a large amount of data and correct only those modules that are computed during the forward pass. After the inference of the mini-batch, we freeze those blocks that have never been activated, and tune the remaining blocks in the process of backward error propagation. The size of a mini-batch can be one or more. Mini-batches with a size greater than one in the forward pass have a computational advantage on the GPU only. A mini-batch size greater than one in the backward pass provides a more accurate gradient estimation. Even for a fixed number of instances for a few-shot problem, the number of adaptation steps can be selected or optimized. In this case, mini-batches can be sampled from a subset of the task with replacement.

Unexpected changes in the domain or other distributional shifts can always occur. A promising approach to mitigate such disturbances is to use the ideas and methods of test-time augmentation and test-time adaptation [15,28]. It is proposed to calculate the entropy of the marginal probability of low confidence predictions generated by the model tuning over a certain number of iterations by analogy with scientific research [10]. The loss function for test-time adaptation to the input exemplar x, which may belong to a certain category from the set Y, will be

(6) $$L_{\mathrm{TTA}}\left(\Psi ,x\right)\approx H\left({\bar{p}}_{\Psi }\left(\cdot |x\right)\right)=\sum_{y\in Y}{\;\bar{p}}_{\Psi }(y|x){\log\;\bar{p}}_{\Psi }\left(y|x\right),$$

where ${\;\bar{p}}_{\Psi }\left(y|x\right)$ is an estimate of the marginal probability at the model output calculated by the formula

(7) $${\;\bar{p}}_{\Psi }\left(y|x\right)=\frac{1}{B}\sum_{i=1}^{B}p_{\Psi }(y|\tilde{x_{i}}),$$

where $\tilde{x_{i}}$ is the i-th augmented version of the input observation.

If, after tuning, the entropy at the model output does not exceed the threshold value, it is necessary to switch to the graceful degradation mode. The easiest way to use graceful degradation is to hand over control to a human to fine-tune the system on labeled samples.

## 4. Experiments and Results

### 4.1. Experimental Setup

The CIFAR-10 and CIFAR-100 datasets were selected for experimentation due to their public availability and the small size of their images, which accelerates experimental research [33]. The CIFAR-10 dataset consists of 50,000 training images and 10,000 test 32 × 32 color images distributed evenly between 10 classes. The CIFAR-100 dataset has 100 classes containing 600 images each. For convenience of the analysis, we will use 70% of training data to form a base dataset for the training of the base model, and use the remaining 30% for the additional training dataset.

Since convolutional architecture and transformer-based architecture are two of the most popular and well-developed architectures in the field of artificial intelligence and machine vision, both architectures are considered in the experiments. The ResNet-110 backbone is used to build a dynamic convolutional network. In such a convolutional network, gate modules control the involvement of ResNet blocks in adaptive computing. The DeiT-S backbone is used to build a dynamic visual transformer. Gate units of the transformer control the involvement of encoders in adaptive computing.

In this case, a set of 10 classes will be used for few-shot learning tasks, which are randomly selected from the set of available classes ($n_{\mathrm{way}}=10$). It is proposed to use 16 images per class (k_shot = 16), which are provided in mini-batches of four images (mini_batch_size = 4) during adaptation. Thus, the number of adaptation steps is T = (k_shot × $n_{\mathrm{way}}$)/mini_batch_size = 20 iterations. The learning rate of the inner and outer loop of meta-learning are α = 0.001 and β = 0.0001, respectively. The maximum number of meta-iterations is 300. The Early Stopping algorithm is used to stop meta-learning, which terminates the execution if the criterion does not change for more than 10 consecutive iterations by more than 0.001.

To enhance the training of AI models, fault injection is implemented by selecting weights with the highest absolute gradient values. The proportion of modified weights is determined by a fault rate of 0.1. During the testing phase, the resulting model undergoes fault injection through random bit-flips in randomly selected weights, maintaining a fault rate of 0.1.

The training of the AI model involves generating adversarial samples from normalized images using the FGSM algorithm, with a perturbation level defined by the L∞ norm up to 3/255. However, to evaluate the robustness of the resulting model against adversarial attacks, the adversarial samples are generated using the CMA-ES algorithm, maintaining a perturbation level of 3/255 according to the L∞ norm.

Considering the elements of randomization, the average values are proposed for assessing the model’s accuracy. For this purpose, 100 instances of a specific type of disturbance are generated and applied to the same model or dataset. The average accuracy during adaptation to new tasks is subsequently estimated based on five task implementations.

After each experiment on adaptation to a certain type of disturbance, we can calculate the value of the integral resilience $R_{\tau_{i}}$ to the implementation of this disturbance $\tau_{i}$

(8) $$R_{\tau_{i}}=\frac{1}{P_{0}T}\sum_{t=1}^{T}P_{\tau_{i}}\left(\Psi ,W_{\tau_{i},t},D_{\tau_{i}}^{\mathrm{val}}\right),$$

where $P_{\tau_{i}}$ is a performance metric for the current state of model parameters and evaluation data;

$P_{0}$ is a model performance before disturbance impact;

T is a maximum number of adaptation steps.

The greater the robustness of the model to the disturbance $\tau_{i}$, the greater the value of $R_{\tau_{i}}$. Likewise, the faster the model recovers or improves its performance during T adaptation steps, the greater the value of $R_{\tau_{i}}$.

### 4.2. Results

The accuracy of the convolutional model trained with zero dynamic compression $t_{k}=1.0$ on test data under normal conditions is 0.87. The accuracy of the model based on a visual transformer trained with zero dynamic compression on test data under normal conditions is 0.90. Let us consider how the accuracy and resilience of an AI system depend on the dynamic compression rate and the impact of disturbances at the stage of pre-training.

Table 1 and Table 2 illustrate the effect of the pre-training of the dynamic neural network with dynamic compression and disturbances on the fault injection test accuracy and resilience criterion values. The average accuracy $\bar{\mathrm{Acc}}$ and the integral resilience indicator $\;\bar{R}$ for the model are evaluated on 100 implementations of fault injection with a fault rate of 0.3. The meta-trained model is adapted using the test-time adaptation and supervised fine-tuning.

An analysis of Table 1 and Table 2 shows that training with fault injection improves both accuracy and resilience compared to training under normal conditions. However, meta-training to optimize resilience gives better results than pre-training with fault injection. In this case, the tables show that increasing the compression to a certain level improves accuracy and resilience, but further compression leads to a degradation in the result. The optimal dynamic compression rate is 0.6, as the accuracy and resilience improve slightly while reducing FLOPs by more than 30%. Compared to the base case of training without dynamic compression, this compression rate for the CNN provides a 2.1% increase in the resilience of the model trained under normal conditions, a 0.9% increase in the resilience of the model trained with fault injection, and a 1.5% increase in the resilience of the meta-trained model based on the results of adaptation to disturbances. Compared to the base case of training with no dynamic compression, this compression rate for the ViT provides a 3.2% increase in the resilience of the model trained under normal conditions, a 1.7% increase in the resilience of the model trained with fault injection, and a 1.8% increase in the resilience of the meta-trained model, based on the results of adaptation to disturbances. The proposed training method, compared to the conventional training method, provides a 3.3% increase in the accuracy of the convolutional network and a 24% increase in the resilience under the influence of fault injection at the optimal compression rate. The proposed training method, compared to the conventional training method, provides an increase in the accuracy of the visual transformer by 4.4%, and a 19.7% increase in the resilience under the influence of fault injection at the optimal compression rate.

Table 3 and Table 4 show the effect of pre-training a dynamic neural network with both dynamic compression and perturbation on the values of the accuracy and resilience criteria when testing an AI system under the influence of adversarial attacks. The average accuracy $\bar{\mathrm{Acc}}$ and the integral resilience indicator $\;\bar{R}$ for the model are evaluated on 100 implementations of adversarial attack with a perturbation level of 3/255, according to the L∞ norm. The meta-trained model is adapted using test-time adaptation and supervised fine-tuning.

An analysis of Table 3 and Table 4 shows that training under adversarial attacks improves both accuracy and resilience compared to training under normal conditions. However, meta-training to optimize resilience gives better results than pre-training under adversarial attacks. At the same time, the tables show that an increase in dynamic compression (reduction of the compression rate) up to a certain level provides an increase in accuracy and resilience, but a further increase leads to a degradation of the result. The optimal dynamic compression rate is 0.6, as the accuracy and resilience improve slightly while reducing FLOPs by more than 30%. Compared to the base case of no dynamic compression (compression rate equal to 1.0), this compression rate for a CNN provides a 3.8% increase in the resilience of the model trained under normal conditions, a 6% increase in the resilience of the model trained with fault injection, and a 1.6% increase in the resilience of the meta-trained model based on the results of adaptation to disturbances. Compared to the base case without dynamic compression, the compression rate for the ViT provides a 2% increase in the resilience of the model trained under normal conditions, an 8% increase in the resilience of the model trained with fault injection, and a 1.3% increase in the resilience of the meta-trained model based on the results of adaptation to disturbances. The proposed training method, compared to the conventional training method, provides a 5.7% increase in the accuracy of the convolutional network and a 16.9% increase in its resilience under the influence of adversarial attacks at the optimal compression rate. The proposed training method, compared to the conventional training method, provides a 5.1% increase in the accuracy of the visual transformer and a 21.6% increase in its resilience under the influence of adversarial attacks at the optimal compression rate.

To evaluate the robustness and speed of adaptation of a pretrained AI system to concept drift, it is proposed to calculate the integral resilience criterion (8) in fine-tuning mode (few-shot learning) on the CIFAR-100 dataset (Table 5).

An analysis of Table 5 allows us to conclude that the visual transformer appeared to be less resilient to changing tasks within a limited number of steps. In both cases, for the convolutional network and the visual transformer, an application of meta-learning to optimize resilience significantly improves resilience to task changes. However, there is also a certain optimal value of the dynamic compression rate which maximizes the resilience of the AI system. In the case of the meta-training of the dynamic network to optimize the resilience, it is possible to obtain a higher resilience index at a higher level of dynamic compression (t = 0.6) than under normal conditions (t = 0.8). Meta-training of the neural network model allows us to obtain an average of 22% higher resilience while simultaneously reducing FLOPs by more than 30%.

Table 6 shows the dependence of the AI system’s resilience on the selected activation function with optimal compression rate. The activation functions ReLU and LeakyReLU6 are considered.

The analysis of Table 6 shows that, in all cases, LeakyReLU6 provides a higher value of resilience compared to the ReLU activation function.

In the experimental research, two adaptation mechanisms, test-time adaptation and supervised fine-tuning, are applied sequentially. In real-world scenarios, supervised fine-tuning is only feasible when manual labeling or another source of feedback is available, whereas test-time adaptation does not require labeled data. However, to understand the contribution of each component, Table 7 presents the resilience indicator when only one adaptation mechanism is used.

An analysis of Table 7 shows that supervised fine-tuning makes the largest contribution to the adaptation process. However, the contribution of test-time adaptation also positively impacts resilience when new data are introduced, though its effect is three to six times smaller than that of supervised fine-tuning.

## 5. Discussion

Similar to the research in [34], the proposed model and method of the AI system provide resilience by introducing additional add-ons and, accordingly, increase the number of parameters. However, in the proposed approach, the add-ons reduce the amount of computation in the testing mode. For example, the additional Gate Unit per Residual Block adds a fixed overhead of 0.015% more floating point operations. Adding gate units to the DeiT-S visual transformer increases the overhead by 0.0022% more floating point operations. In this case, a dynamic compression rate of 0.6 allows you to reduce more than 30% FLOPs during inference.

Dynamic inference enables a reduction in FLOPs during inference to the level of smaller neural networks, while achieving higher accuracy and robustness. For example, in the study in [31], a ResNet-50 neural network requiring 130 MFLOPs achieved an accuracy of 60.73% under the influence of a CMA-ES adversarial attack with a perturbation level of 3/255, according to the L∞ norm. According to Table 3, the ResNet-110 neural network with dynamic gate units, and trained under normal conditions with a dynamic compression rate of 0.2, requires an average of 102.8 MFLOPs and achieves an accuracy of 75.4% under the influence of perturbations of the same type and amplitude. If adversarial training based on the FGSM algorithm is applied to this network, the accuracy can be increased to 80.1% under the influence of a CMA-ES adversarial attack with a perturbation level of 3/255, according to the L∞ norm. By applying the proposed resilience-aware meta-learning method, accuracy can be increased to 87.9%, with an average computational cost of 104.69 MFLOPs per observation. In [35], the ResNet-18 network, unaffected by disturbances, achieved an accuracy of 62.66% and required 192.3 MFLOPs of computation, while the ResNet-110 with a compression ratio of 0.2 required 103.10 MFLOPs of computation and achieved an accuracy of 73.4% under the influence of fault injection through random bit-flips.

In addition to speeding up computations, it is possible to increase resilience and accuracy in the face of disturbances. This is probably due to the switching off of a certain part of the vulnerable and irrelevant parts of the neural network. Similarly, the ability of the early exit mechanism to increase fault tolerance and adversarial robustness was considered in [22,24]. However, early exit networks mandatorily compute the lower layers, and the percentage of activated network layers can vary quite a bit. This limits the resiliency and computational efficiency of the AI system under this approach. Gate units provide approximately the same number of activated layers by specifying the dynamic compression rate during training. Experiments have shown that an excessive increase in compression leads to a decrease in both accuracy and resolution. Reducing the number of parameters should lead to an increase in the speed of tuning to adapt to changes. However, it seems that the information capacity of a compressed network is not always enough to ensure resilience to disturbances. That is, there is a certain optimal compression rate at which the accuracy, resiliency, and computational efficiency of the AI system increase simultaneously. In addition, it is possible to determine what losses in accuracy and resilience will occur if the required level of computational costs is achieved.

It has been experimentally confirmed [34] that meta-learning based on the results of adaptation to disturbances provides an increase in robustness compared to conventional learning under the influence of disturbances. However, this paper considers a broader concept of resilience, which includes the ability to adapt quickly. In [18,19], to reduce the resource intensity of adaptation, Parameter-Efficient Transfer Learning Principles were used, that is, only adapters were adapted, and the basic model was unchanged. However, this is more relevant for very large general-purpose models. Our research experimentally confirmed the effectiveness of tuning only a part of the layers that were activated during the forward pass. Despite disturbances simultaneously affecting both the backbone network and gate units, meta-learning has increased the ability to absorb disturbances and the ability to adapt quickly in a few-shot learning scenario.

## 6. Conclusions

### 6.1. Summary

A new resource-efficient model for resilient AI system was proposed. The proposed model included gate units to implement a dynamic neural network. The LeakyReLU6 function was used to increase the efficiency of functioning in the conditions of disturbances in the training and inference modes. The model can deactivate irrelevant blocks both during forward pass and backward propagation when adapting to a disturbance to reduce the resource consumption.

The new training method included offline meta-training on the results of adaptation to disturbances of a dynamic neural network and online adaptation on a small number of labeled and unlabeled examples. In this case, online tuning was performed only for those modules that were activated during the forward pass.

Experimental results obtained from the Cifar10 and Cifar100 datasets showed that the proposed approach provides resource-efficient resilience capabilities to fault injections, concept drifts, and adversarial attacks. In the experiments, ResNet-110 and DeiT-S neural networks were used, and for both, the optimal dynamic compression rate was found to be 0.6, as the accuracy and resilience improved slightly while reducing FLOPs by more than 30%. The proposed training method, compared to the conventional training method, provided a 3.3% increase in the accuracy of the convolutional network and a 24% increase in the resilience indicator under the influence of fault injection (random bit-flips in randomly selected 10% weights) with an optimal compression rate. At the optimal compression rate, the proposed training method, compared to the conventional training method, provided a 4.4% increase in the accuracy of the visual transformer and a 19.7% increase in resilience under the influence of fault injection (random bit-flips in randomly selected 10% weights). The proposed training method, compared to the conventional training method, improved the accuracy of the convolutional network by 5.7% and the resilience indicator by 16.9% under the influence of adversarial attacks (CMA-ES adversarial attack with a perturbation level of 3/255 according to the L∞ norm) with optimal compression rate. The proposed training method, compared to the conventional training method, improved the accuracy of the visual transformer by 5.1% and the resilience indicator by 21.6% under the influence of adversarial attacks with the optimal compression rate. In addition, proposed meta-pretraing to optimize the resilience indicator significantly improved resilience to task changes. Meta-training of the neural network model allowed us to obtainin an average of 22% higher resilience while simultaneously reducing FLOPs by more than 30%.

The practical significance of the research derives from a new methodological basis for the development of AI system with affordable resilience to adversarial attacks, faults, and concept drifts under resource constraints.

### 6.2. Limitations

This research demonstrates a way to reduce computational complexity while increasing the resilience on the example of the image classification task. However, other types of machine learning tasks were not considered, as it is assumed that the effectiveness of this approach is task-agnostic.

Moreover, the study was limited to the consideration of a convolutional neural network and a visual transformer. The impact of different gate unit architectures on resiliency was not investigated. Different disturbance intensities were not investigated. Also, the effect of the compression rate on the size of the initial network was not considered.

### 6.3. Future Research Directions

Future research should focus on studying the effect of network size and architecture on the efficiency and resilience of a dynamic neural network that is meta-trained on the results of adaptation to disturbances. In addition, an important direction of research is the development of resilient AI systems for various applications based on the principle of proactivity [36], which can be implemented at the component and system levels.

## Figures and Tables

**Table 1:** Experimental data of ResNet model resilience to the fault injection testing.

Dynamic Compression Rate	Pretrained Model under Normal Condition			Pretrained Model under Fault Injection			Pretrained Model Optimized for Resilience		
	MFLOPs	$\bar{\mathrm{Acc}}$	$\bar{R}$	MFLOPs	$\bar{\mathrm{Acc}}$	$\bar{R}$	MFLOPs	$\bar{\mathrm{Acc}}$	$\bar{R}$
1.0	510.48	85.1%	0.774	510.48	87.5%	0.944	510.48	89.1%	0.973
0.8	408.56	86.5%	0.792	410.44	87.6%	0.949	416.11	89.2%	0.986
0.6	306.64	87.0%	0.791	310.66	87.9%	0.953	312.30	89.9%	0.988
0.4	204.72	81.7%	0.771	206.55	85.5%	0.950	208.52	89.1%	0.975
0.2	102.80	75.4%	0.694	103.33	80.1%	0.942	104.69	87.9%	0.957

**Table 2:** Experimental data of DeiT-S model’s resilience to the fault injection.

Dynamic Compression Rate	Pretrained Model under Normal Condition			Pretrained Model under Fault Injection			Pretrained Model Optimized for Resilience		
	GFLOPs	$\bar{Acc}$	$\bar{R}$	GFLOPs	$\bar{Acc}$	$\bar{R}$	GFLOPs	$\bar{Acc}$	$\bar{R}$
1.0	10.56	86.3%	0.763	10.56	88.1%	0.933	10.56	90.6%	0.961
0.8	7.92	87.2%	0.781	8.11	88.8%	0.941	8.80	91.1%	0.970
0.6	6.16	88.3%	0.788	6.55	89.2%	0.949	7.04	92.2%	0.979
0.4	3.52	84.6%	0.764	4.07	87.1%	0.943	4.40	90.5%	0.965
0.2	1.76	77.2%	0.688	1.98	83.3%	0.931	2.64	88.8%	0.944

**Table 3:** Experimental data of the ResNet-110 model’s resilience to the adversarial attack testing.

Dynamic Compression Rate	Pretrained Model under Normal Condition			Pretrained Model under Adversarial Attack			Pretrained Model Optimized for Resilience		
	MFLOPs	$\bar{Acc}$	$\bar{R}$	MFLOPs	$\bar{Acc}$	$\bar{R}$	MFLOPs	$\bar{Acc}$	$\bar{R}$
1.0	510.48	83.1%	0.754	510.48	85.5%	0.801	510.48	88.1%	0.902
0.8	409.99	83.5%	0.772	412.13	86.1%	0.822	424.01	88.2%	0.910
0.6	309.71	84.0%	0.784	312.01	86.8%	0.853	319.54	88.8%	0.917
0.4	206.88	75.7%	0.781	207.43	84.4%	0.850	208.52	87.4%	0.905
0.2	103.10	73.4%	0.685	103.98	78.4%	0.841	104.55	87.1%	0.888

**Table 4:** Experimental data of the DeiT-S model’s resilience to the adversarial attack testing.

Dynamic Compression Rate	Pretrained Model under Normal Condition			Pretrained Model under Adversarial Attack			Pretrained Model Optimized for Resilience		
	GFLOPs	$\bar{Acc}$	$\bar{R}$	GFLOPs	$\bar{Acc}$	$\bar{R}$	GFLOPs	$\bar{Acc}$	$\bar{R}$
1.0	10.56	85.2%	0.744	10.56	85.9%	0.781	10.56	88.9%	0.911
0.8	7.92	85.7%	0.751	8.61	86.8%	0.812	9.42	88.9%	0.923
0.6	6.50	85.7%	0.759	6.95	87.1%	0.844	7.26	90.1%	0.923
0.4	3.22	77.6%	0.731	3.87	86.3%	0.839	4.22	88.5%	0.920
0.2	1.76	75.0%	0.705	1.88	80.7%	0.833	2.24	88.1%	0.914

**Table 5:** The value of the integral resilience criterion (8) to the change of image analysis task depending on the dynamic compression rate.

Dynamic Compression Rate	Fine-Tuning of ResNet-110-Based AI System		Fine-Tuning of DeiT-S-Based AI System	
	Pretrained on the Base Dataset under Normal Condition	Meta-Trained on Result of Adaptation to Disturbances for Resilience Optimization	Pretrained on Base Dataset under Normal Condition	Meta-Trained on Result of Adaptation to Disturbances for Resilience Optimization
1.0	0.791	0.962	0.761	0.921
0.8	0.809	0.978	0.762	0.930
0.6	0.805	0.988	0.759	0.935
0.4	0.761	0.955	0.732	0.902
0.2	0.731	0.892	0.711	0.803

**Table 6:** The value of the integral resilience criterion (8) depending on the activation function, network type, and type of disturbance with optimal compression rate.

Disturbance Type	Fine-Tuning of ResNet-110-Based AI System		Fine-Tuning of DeiT-S-Based AI System	
	ReLU	LeakyReLU6	ReLU	LeakyReLU6
Fault Injection	0.953	0.988	0.950	0.979
Adversarial Attack	0.891	0.917	0.884	0.923
Task Change	0.978	0.988	0.911	0.935

**Table 7:** The value of the integral resilience criterion (8) depending on partial fine-tuning setting with optimal compression rate.

Meta-Trained Backbone	Fine-Tuning Setting	${\bar{R}}^{-}↓$ (%) under Adversarial Attack	${\bar{R}}^{-}↓$ (%) under Fault Injection Attack
ResNet-110	Only Supervised Fine-Tuning	3.1	2.9
	Only Test-Time-Adaptation	9.7	11.3
DeiT-S	Only Supervised Fine-Tuning	2.6	1.9
	Only Test-Time-Adaptation	11.7	10.5

## Data Availability

The CIFAR-10 and CIFAR-100 datasets presented in this study are openly available in [27] and can be found here https://www.cs.toronto.edu/~kriz/cifar.html, accessed on 17 September 2022. The ILSVRC2012 dataset can be found here http://image-net.org/challenges/LSVRC/2012/index, accessed on 17 September 2022.

## References

[1] Li Z., Li H., Meng L. (2023). Model Compression for Deep Neural Networks: A Survey. Computers.

[2] Marinó G.C., Petrini A., Malchiodi D., Frasca M. (2023). Deep neural networks compression: A comparative survey and choice recommendations. Neurocomputing.

[3] Moskalenko V., Kharchenko V., Moskalenko A., Kuzikov B. (2023). Resilience and Resilient Systems of Artificial Intelligence: Taxonomy, Models and Methods. Algorithms.

[4] Olowononi F.O., Rawat D.B., Liu C. (2021). Resilient Machine Learning for Networked Cyber Physical Systems: A Survey for Machine Learning Security to Securing Machine Learning for CPS. IEEE Commun. Surv. Tutor..

[5] Samangouei P., Kabkab M., Chellappa R. (2018). Defense-GAN: Protecting Classifiers Against Adversarial Attacks Using Generative Models (Version 2). arXiv.

[6] Hussain M., Hong J.-E. (2023). Reconstruction-Based Adversarial Attack Detection in Vision-Based Autonomous Driving Systems. Mach. Learn. Knowl. Extr..

[7] Ho J., Jain A., Abbeel P. (2020). Denoising Diffusion Probabilistic Models (Version 2). arXiv.

[8] Sooksatra K., Hamerly G., Rivas P. Is ReLU Adversarially Robust? [Poster Presentation]. Proceedings of the Computer Vision and Pattern Recognition Conference: LatinX in AI (LXAI) Research Workshop 2023.

[9] Hou X., Breier J., Jap D., Ma L., Bhasin S., Liu Y. (2020). Security Evaluation of Deep Neural Network Resistance Against Laser Fault Injection. Proceedings of the 2020 IEEE International Symposium on the Physical and Failure Analysis of Integrated Circuits (IPFA).

[10] Guo Y., Li S., Lerman G. (2024). The effect of Leaky ReLUs on the training and generalization of overparameterized networks. Proc. Mach. Learn. Res..

[11] Cavagnero N., Santos F.D., Ciccone M., Averta G., Tommasi T., Rech P. Transient-Fault-Aware Design and Training to Enhance DNNs Reliability with Zero-Overhead. Proceedings of the 2022 IEEE 28th International Symposium on On-Line Testing and Robust System Design (IOLTS).

[12] Niu Z., Chen Z., Li L., Yang Y., Li B., Yi J. (2020). On the Limitations of Denoising Strategies as Adversarial Defenses. arXiv.

[13] Eleftheriadis C., Symeonidis A., Katsaros P. (2024). Adversarial robustness improvement for deep neural networks. Mach. Vis. Appl..

[14] Sum J., Leung C.-S. (2023). Regularization Effect of Random Node Fault/Noise on Gradient Descent Learning Algorithm. IEEE Trans. Neural Netw. Learn. Syst..

[15] Zhang M., Levine S., Finn C. (2022). MEMO: Test Time Robustness via Adaptation and Augmentation. arXiv.

[16] Son X., Yang Y., Choromanski K., Caluwaerts K., Gao W., Finn C., Tan J. Rapidly adaptable legged robots via evolutionary meta-learning. Proceedings of the 2020 IEEE/RSJ International Conference on Intelligent Robots and Systems (IROS).

[17] Wang R., Xu K., Liu S., Chen P.-Y., Weng T.W., Gan C., Wang M. (2021). On Fast Adversarial Robustness Adaptation in Model-Agnostic Meta-Learning. arXiv.

[18] Ding N., Qin Y., Yang G., Wei F., Yang Z., Su Y., Hu S., Chen Y., Chan C.M., Chen W. (2023). Parameter-efficient fine-tuning of large-scale pre-trained language models. Nat. Mach. Intell..

[19] Asadi N., Beitollahi M., Khalil Y., Li Y., Zhang G., Chen X. (2024). Does Combining Parameter-efficient Modules Improve Few-shot Transfer Accuracy? (Version 1). arXiv.

[20] Wang M., Mo J., Lin J., Wang Z., Du L. DynExit: A Dynamic Early-Exit Strategy for Deep Residual Networks. Proceedings of the 2019 IEEE International Workshop on Signal Processing Systems (SiPS).

[21] Veit A., Belongie S. Convolutional Networks with Adaptive Inference Graphs. Proceedings of the Computer Vision—ECCV 2018.

[22] Haque M., Yang W. Dynamic Neural Network is All You Need: Understanding the Robustness of Dynamic Mechanisms in Neural Networks. Proceedings of the 2023 IEEE/CVF International Conference on Computer Vision Workshops (ICCVW).

[23] Moskalenko V., Moskalenko A. (2022). Neural network based image classifier resilient to destructive perturbation influences—Architecture and training method. Radioelectron. Comput. Syst..

[24] Wang J., Zhang Z., Wang M., Qiu H., Zhang T., Li Q., Li Z., Wei T., Zhang C. (2023). Aegis: Mitigating Targeted Bit-flip Attacks against Deep Neural Networks (Version 1). arXiv.

[25] Peng Y., Lee J., Watanabe S. I3D: Transformer Architectures with Input-Dependent Dynamic Depth for Speech Recognition. Proceedings of the ICASSP 2023—2023 IEEE International Conference on Acoustics, Speech and Signal Processing (ICASSP).

[26] Meng L., Li H., Chen B.-C., Lan S., Wu Z., Jiang Y.-G., Lim S.-N. AdaViT: Adaptive Vision Transformers for Efficient Image Recognition. Proceedings of the 2022 IEEE/CVF Conference on Computer Vision and Pattern Recognition (CVPR).

[27] Shen T., Lee C., Narayanan V. Multi-Exit Vision Transformer with Custom Fine-Tuning for Fine-Grained Image Recognition. Proceedings of the 2023 IEEE International Conference on Image Processing (ICIP).

[28] Moskalenko V., Kharchenko V. (2024). Resilience-aware MLOps for AI-based medical diagnostic system. Front. Public Health.

[29] Gharoun H., Momenifar F., Chen F., Gandomi A.H. (2023). Meta-learning approaches for few-shot learning: A survey of recent advances (Version 1). arXiv.

[30] Bortsova G., González-Gonzalo C., Wetstein S.C., Dubost F., Katramados I., Hogeweg L. (2021). Adversarial attack vulnerability of medical image analysis systems: Unexplored factors. Med. Image Anal..

[31] Kotyan S., Vargas D.V. (2022). Adversarial robustness assessment: Why in evaluation both L0 and L∞ attacks are necessary. PLoS ONE.

[32] Li G., Pattabiraman K., DeBardeleben N. TensorFI: A Configurable Fault Injector for TensorFlow Applications. Proceedings of the 2018 IEEE International Symposium on Software Reliability Engineering Workshops (ISSREW).

[33] Foldy-Porto T., Venkatesha Y., Panda P. Activation Density Driven Efficient Pruning in Training. Proceedings of the 2020 25th International Conference on Pattern Recognition (ICPR).

[34] Moskalenko V.V. (2023). Model-Agnostic Meta-Learning for Resilience Optimization of Artificial Intelligence System. Radio Electron. Comput. Sci. Control.

[35] Chen Z., Narayanan N., Fang B., Li G., Pattabiraman K., DeBardeleben N. TensorFI: A Flexible Fault Injection Framework for TensorFlow Applications. Proceedings of the 2020 IEEE 31st International Symposium on Software Reliability Engineering (ISSRE).

[36] Lysenko S., Kharchenko V., Bobrovnikova K., Shchuka R. (2020). Computer systems resilience in the presence of cyber threats: Taxonomy and ontology. Radioelectron. Comput. Syst..

